# Mechanisms Underlying Hyperexcitability: Combining Mossy Fiber Sprouting and Mossy Cell Loss in Neural Network Model of the Dentate Gyrus

**DOI:** 10.3390/biomedicines13061416

**Published:** 2025-06-09

**Authors:** Dariusz Świetlik

**Affiliations:** Division of Biostatistics and Neural Networks, Medical University of Gdansk, Debinki 1 St., 80-211 Gdansk, Poland; dariusz.swietlik@gumed.edu.pl

**Keywords:** epilepsy, dentate gyrus, mossy fiber sprouting, mossy cell loss, networks model

## Abstract

**Background/Objectives**: A concussive head injury increases the likelihood of temporal lobe epilepsy through mechanisms that are not entirely understood. This study aimed to investigate how two key histopathological features shared by both TLE (temporal lobe epilepsy) and head injury—mossy fiber sprouting and hilar excitatory cell loss—contribute to the modulation of dentate gyrus excitability. **Methods**: A computational approach was used to explore the impact of specific levels of mossy fiber sprouting and mossy cell loss, while avoiding the confounding effects of concurrent changes. The dentate gyrus model consists of 500 granule cells, 15 mossy cells, 6 basket cells and 6 hilar perforant path-associated cells. **Results**: My simulations demonstrate a correlation between the degree of mossy fiber sprouting and the number of spikes in dentate gyrus granule cells (correlations coefficient R = 0.95, *p* < 0.0001) and other cells (correlations coefficient R = 0.99, *p* < 0.0001). The mean values (standard deviation, SD) and 95% CI for granule cell activity in the control group and percentage 10–50% of mossy fiber sprouting groups are 376.4 (16.7) (95% CI, 374.9–377.8) vs. 463.5 (24.3) (95% CI, 461.4–465.6) vs. 514.8 (32.5) (95% CI, 511.9–517.6) vs. 555.0 (40.4) (95% CI, 551.5–558.6) vs. 633.4 (51.8) (95% CI, 628.8–637.9) vs. 701.7 (66.2) (95% CI, 695.9–707.5). The increase in mossy fiber sprouting was significantly statistically associated with an increase in granule cell activity (*p* < 0.01). The removal of mossy cells led to a reduction in excitability within the model network (for granule cells, correlations coefficient R = −0.40, *p* < 0.0001). **Conclusions**: These results are generally consistent with experimental observations, which indicate a high degree of mossy fiber sprouting in animals with a higher frequency of seizures. Whereas unlike the strong hyperexcitability effects induced by mossy fiber sprouting, the removal of mossy cells led to reduced granule cell responses to perforant path activation.

## 1. Introduction

The precise mechanisms responsible for post-traumatic hyperexcitability in the dentate gyrus (DG) remain incompletely understood; however, experimental head trauma is associated with a distinct pattern of cell loss in the hilus [1,2,3]. More recently, whole-brain connectomics has revealed that traumatic brain injury profoundly rewires dentate inhibitory circuits and destabilizes mossy cell populations, offering a network-level explanation for chronic DG hyperexcitability [4]. Moreover, experimental head injury is linked to the development of recurrent excitatory collateral sprouting from granule cell axons [5,6]. These changes resemble the histopathological features found in tissue from individuals with temporal lobe epilepsy, where currently around 50 million individuals are affected by epilepsy, with approximately 40% of these cases classified as temporal lobe epilepsy [7,8].

Contemporary work corroborates these observations: silencing DG activity mitigates MFS and delays epileptogenesis in kindling models [9], while high-resolution studies link MeCP2 signaling [10] and adult-born granule cells to injury-induced axon sprouting [11].

Although MFS is widely thought to amplify excitatory feedback within the granule-cell layer, its exact role in seizure generation remains debated. Emerging data suggest a dorsoventral imbalance in mossy-cell vulnerability and context-dependent modulation of dentate gating [7,12,13,14,15]. Mossy fibers typically extend through the dentate hilus and stratum lucidum, forming synaptic connections with hilar cells and CA3 neurons [16]. In the hippocampus of individuals with temporal lobe epilepsy, however, mossy fiber collaterals aberrantly extend into the inner molecular layer of the dentate gyrus, where the sprouted fibers provide excitatory recurrent input to the dendrites of granule cells [17,18]. This reorganization is commonly believed to contribute to hippocampal hyperexcitability, although the precise role of mossy fiber sprouting in TLE remains a subject of research.

Thus, the complexity of investigating the primary mechanisms underlying seizure onset makes computational simulation of the dentate gyrus an essential tool for gaining new insights. Computer modeling [19,20,21,22] has facilitated significant progress in understanding complex phenomena, such as epilepsy [23,24,25,26,27,28], and in silico studies have been effectively utilized to reduce, refine, and partially substitute for animal and human experimentation [29,30,31]. The study extends classical Fickian diffusion by introducing concentration-dependent coefficients and a memory kernel, yielding a spatiotemporal equation that more closely matches experimentally observed calcium spread inside cells. Its simulations show that incorporating cellular “memory” produces non-Gaussian diffusion fronts and markedly slower equilibration, providing a mechanistic explanation for heterogeneous calcium microdomains [32]. Complementing this macroscopic perspective, the paper applies artificial neural networks to model local neuronal calcium transients, demonstrating that data-driven architectures can capture nonlinear channel kinetics and outperform traditional deterministic models in predicting sub-micron signaling hotspots [33].

As demonstrated earlier, modeling analysis serves as a valuable tool for investigating the epileptogenic mechanisms underlying temporal lobe epilepsy. A comprehensive understanding of the mechanisms underlying the generation and propagation of TLE within the hippocampal-entorhinal cortex system requires an integrated network model that emphasizes neuronal diversity. Accordingly, I have developed a neuronal network model to investigate the fundamental mechanisms underlying epilepsy generation. The simulation model integrates formalisms derived from previous studies [19,20,21].

This study aimed to deepen mechanistic understanding of the potential roles of mossy fiber sprouting and mossy cell loss in dentate excitability, with a specific focus on post-traumatic hyperexcitability. The simulations enable discussion on the intricate role of synaptic reorganization associated with experimental models of status epilepticus.

## 2. Materials and Methods

**Figure 1:**
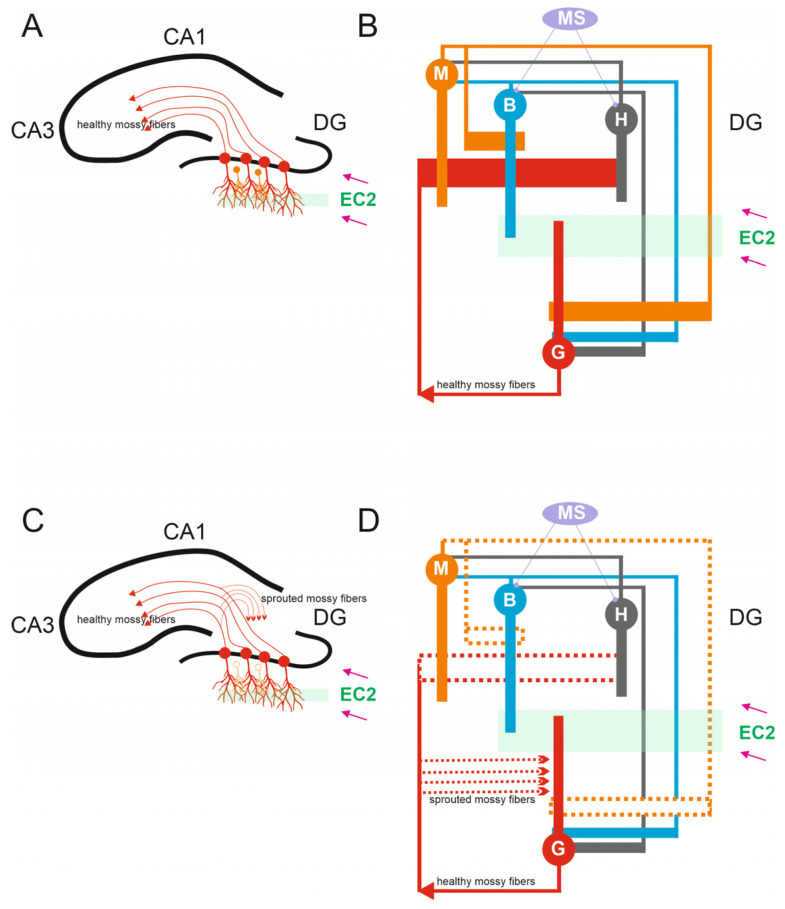
Anatomy of Dentate Circuits. (**A**) Schematic of hippocampal structures including the DG, CA3, and CA1. In a healthy brain, mossy fibers are the axons that arise from glutamatergic dentate granule cells found within the granule cell layer of the dentate gyrus. Healthy mossy fibers provide input to CA3 pyramidal cells. DG granule cells received excitatory inputs from the entorhinal cortex 2 (EC2). (**B**) A representation of the simulated microcircuit model of the DG network, granule cells G, mossy cells M, basket cells B, hilar perforant path-associated cells H, and medial-septum-diagonal band (MS). (**C**) In the epileptic hippocampus, the loss of mossy fiber targets in the hilus leads granule cell axons to sprout and densely innervate the inner molecular layer of the dentate gyrus, a process known as mossy fiber sprouting. (**D**) In the case of mossy cell loss, chosen mossy cells were effectively “killed” by removing all synapses linked to and from these “dead” cells (dashed line).

A scaled-down version of the dentate network was developed, reducing the number of the four primary cell types in the dentate gyrus by a factor of 2000:1 [34]. Figure 1 displays the structures of the hippocampus, such as the dentate gyrus, CA3, CA1, and the representation of the simulated microcircuit model for the DG network. The dentate gyrus model consists of 500 granule cells, 15 mossy cells, 6 basket cells and 6 hilar perforant path-associated cells. Based on their morphological structure and the presence of specific markers, neurons in the dentate gyrus are categorized into excitatory and inhibitory neurons. The first two neuron types are of the excitatory kind, whereas the latter two are inhibitory. The simulation model incorporated formalism from earlier studies [19,20,21]. A simplified demo version of the simulation model is available on the website: https://github.com/dswietlik/Dariusz-Swietlik/blob/main/Neuron%20model (accessed on 17 February 2022). The simulation environment was developed using the Delphi programming language. Each simulation consisted of 20,000 steps, with a temporal resolution of 0.5 milliseconds per step. The duration of each simulation is 10 s.

### 2.1. Development of Biophysically Accurate Multicompartmental Models of Dentate Cells

We developed a multi-compartment hippocampal network model comprising dentate granule cells, basket interneurons and mossy cells. Each cell type was represented with simplified morphologies that included the soma, dendrite, and axon. The synaptic scheme incorporates AMPA- and NMDA-mediated glutamatergic transmission alongside GABAergic inhibition. Model equations and connectivity rules follow previously validated formalisms. Neuronal morphologies were reduced to 16 biophysical compartments—soma, a proximal axonal segment and simplified dendritic trees—whose passive and active properties were drawn from experimental and computational studies [19,20,21]. Excitatory and inhibitory synapses were assigned to the appropriate compartments. To capture intrinsic rhythmic drive, we imposed septo-hippocampal theta input (4–12 Hz), transmitted via the fornix and phase-locked to faster gamma oscillations, consistent with in vivo observations.

The biophysical characteristics of each cell were derived from cell types documented in the literature, which have been thoroughly validated against experimental data [17,23,35,36,37,38,39,40]. Table 1 provides details showing that the intrinsic properties of the model cells depicted in Figure 1 align with data from their biological equivalents in ionotropic receptors [3,41].

### 2.2. Granule Cells

Every granule cell comprised 16 compartments, each equipped with a calcium pump, a sodium current, and currents for K^+^ and Ca^2+^. Every granule cell received excitatory input on its distal third dendrites from layer 2 of the Entorhinal Cortex (EC), and on its proximal third dendrites from mossy cells. Additionally, it received inhibitory inputs on its soma and medial third dendrites from basket cells and hilar perforant path-associated cells.

### 2.3. Mossy Cells

Each mossy cell consisted of 16 compartments, each containing Na^+^, K^+^ and Ca^2+^ currents. Each mossy cell received excitatory inputs in its proximal dendrites from granule cells and other mossy cells, along with inhibitory inputs in its soma and medial dendrites from the basket cells and hilar perforant path-associated cell.

### 2.4. Basket Cells

Each basket cell consisted of 16 compartments, each containing Na^+^ and K^+^ currents. Each basket cell received excitatory input from layer 2 of the Entorhinal Cortex, excitatory inputs from granule and mossy cells in its proximal and medial dendrites, inhibitory inputs to its soma from medial septum, and inhibitory inputs from hilar perforant path-associated cells and other basket cells.

### 2.5. Hilar Perforant Path-Associated Cells

Hilar perforant path-associated cells consisted of 16 compartments, each containing Na^+^ and K^+^ currents. Each hilar perforant path-associated cell received excitatory input from granule cells in its proximal dendrites, excitatory inputs from the mossy cells in its medial dendrites, and inhibitory input to its soma from the medial septum.

### 2.6. Simulating Mossy Fiber Sprouting

Synaptic connections from granule cells to the proximal dendrites of other granule cells were introduced to simulate mossy fiber sprouting. In our scaled-down network, we postulated that 100 newly sprouted synapses equated to 100% mossy fiber sprouting [23]. In this study, the density of mossy fiber sprouting was limited to 0–50% to model the mild to moderate increase in mossy fiber sprouting after a concussive head injury [5]. It should be noted that 100% sprouting corresponds to the level of sprouting seen following seizures induced by pilocarpine [17].

### 2.7. Mossy Cell Loss

To simulate total loss of mossy cells, all synaptic connections to and from mossy cells were eliminated. In the case of 50% mossy cell loss, eight randomly chosen mossy cells were effectively “killed” by removing all synapses linked to and from these “dead” cells [23].

### 2.8. Statistical Methods

Statistical evaluations were conducted using Statistica TIBCO Software Inc., San Ramon, CA, USA (2017) (data analysis software system), version 13. https://www.tibco.com/. ANOVA or the Kruskal–Wallis test was used to demonstrate differences between groups along with post hoc tests. In order to determine dependence, strength, and direction between variables, correlation analysis was used by determining Spearman’s correlation coefficients. The significance level was set at 5%.

## 3. Results

### 3.1. The Role of Pathological Phenomena of Mossy Fiber Sprouting

**Figure 2:**
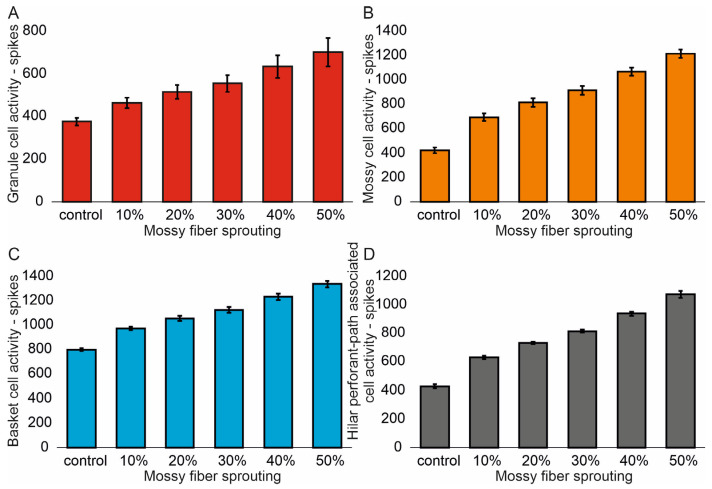
The cell activity (spikes) in the control group and mossy fiber sprouting groups. (**A**) Granule cell activity. (**B**) Mossy cell activity. (**C**) Basket cell activity. (**D**) Hilar perforant path-associated cell activity.

The mean values (standard deviation, SD) and 95% CI for granule cell activity in the control group and percentage 10–50% of mossy fiber sprouting groups are 376.4 (16.7) (95% CI, 374.9–377.8) vs. 463.5 (24.3) (95% CI, 461.4–465.6) vs. 514.8 (32.5) (95% CI, 511.9–517.6) vs. 555.0 (40.4) (95% CI, 551.5–558.6) vs. 633.4 (51.8) (95% CI, 628.8–637.9) vs. 701.7 (66.2) (95% CI, 695.9–707.5). There were statistically significant differences in granule cell activity versus groups (*p* < 0.001). Post hoc tests showed that granule cell activity was significantly higher in mossy fiber sprouting groups relative to the control group (*p* < 0.01). The increase in mossy fiber sprouting was significantly statistically associated with an increase in granule cell activity (*p* < 0.01), Figure 2A. A correlation was obtained between the degree of mossy fiber sprouting and the number of spikes for granule cells (correlations coefficient R = 0.95, *p* < 0.0001). The mean values (standard deviation, SD) and 95% CI for mossy cell activity in the control group and percentage 10–50% of mossy fiber sprouting groups are 424.5 (23.7) (95% CI, 411.4–437.7) vs. 695.7 (29.7) (95% CI, 679.2–712.1) vs. 815.7 (34.8) (95% CI, 796.5–835.0) vs. 915.7 (35.0) (95% CI, 896.3–935.0) vs. 1069.3 (33.1) (95% CI, 1051.0–1087.7) vs. 1216.6 (33.0) (95% CI, 1198.3–1234.9). There were statistically significant differences in mossy cell activity versus groups (*p* < 0.01). The increase in mossy fiber sprouting was significantly statistically associated with an increase in mossy cell activity (*p* < 0.0001), similar for granule cells Figure 2B. A correlation was obtained between the degree of mossy fiber sprouting and the number of spikes for mossy cells (correlations coefficient R = 0.99, *p* < 0.0001).

The mean values (standard deviation, SD) and 95% CI for basket cell activity in the control group and percentage 10–50% of mossy fiber sprouting groups are 799.3 (12.2) (95% CI, 786.5–812.2) vs. 974.2 (14.9) (95% CI, 958.6–989.8) vs. 1056.2 (21.4) (95% CI, 1033.7–1078.7) vs. 1124.5 (25.3) (95% CI, 1097.9–1151.1) vs. 1232.3 (24.5) (95% CI, 1206.6–1258.0) vs. 1336.3 (27.3) (95% CI, 1307.7–1365.0). There were statistically significant differences in basket cell activity versus groups (*p* < 0.0001). The increase in mossy fiber sprouting was significantly statistically associated with an increase in basket cell activity (*p* < 0.0001), Figure 2C. A correlation was obtained between the degree of mossy fiber sprouting and the number of spikes for basket cells (correlations coefficient R = 0.99, *p* < 0.0001). The mean values (standard deviation, SD) and 95% CI for hilar perforant path-associated cell activity in the control group and percentage 10–50% of mossy fiber sprouting groups are 429.3 (13.5) (95% CI, 415.2–443.5) vs. 629.3 (12.2) (95% CI, 616.5–642.1) vs. 731.8 (8.5) (95% CI, 722.9–740.8) vs. 815.8 (11.8) (95% CI, 803.4–828.2) vs. 939.8 (14.2) (95% CI, 924.9–954.7) vs. 1074.5 (23.5) (95% CI, 1049.8–1099.2). There were statistically significant differences in hilar perforant path-associated cell activity versus groups (*p* < 0.0001). Post hoc tests showed that hilar perforant path-associated cell activity were significantly higher in 30–50% mossy fiber sprouting groups, relative to the control group (*p* < 0.05). Furthermore, post hoc tests showed that hilar perforant path-associated cell activity was significantly higher in 40–50% mossy fiber sprouting groups relative to the 10% mossy fiber sprouting group (*p* < 0.05) and were significantly higher in 50% mossy fiber sprouting group relative to 20% mossy fiber sprouting group (*p* = 0.0463) Figure 2D. A correlation was obtained between the degree of mossy fiber sprouting and the number of spikes for hilar perforant path-associated cells (correlations coefficient R = 0.99, *p* < 0.0001).

**Figure 3:**
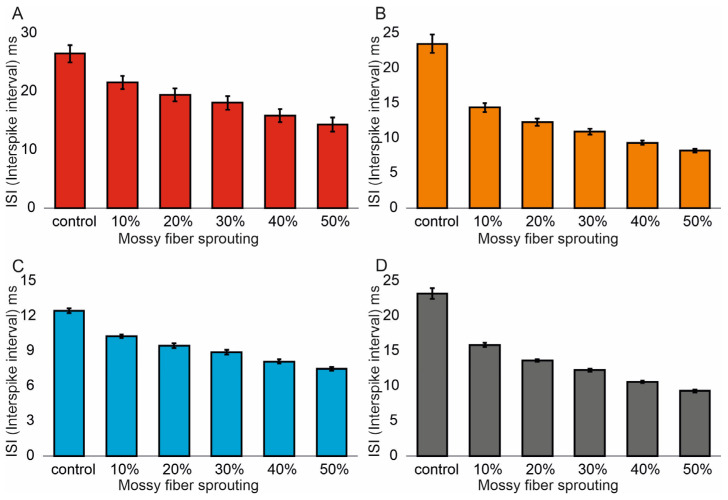
Interspike interval in the control group and mossy fiber sprouting groups. (**A**) Granule cell activity. (**B**) Mossy cell activity. (**C**) Basket cell activity. (**D**) Hilar perforant path-associated cell activity.

Similar associations were obtained for ISI (Interspike interval). In the control model for granule cells, the value was 26.5 (1.4) ms and (95% CI, 26.4–26.6). However, with increasing mossy fiber sprouting, the values were 21.6 (1.1) ms and (95% CI, 21.5–21.7), 19.5 (1.1) ms and (95% CI, 19.4–19.6), 18.1 (1.2) ms and (95% CI, 18.0–18.2), 15.9 (1.1) ms and (95% CI, 15.8–16.0), and 14.3 (1.2) ms and (95% CI, 14.2–14.4). There were statistically significant ISI differences for granule cells with increasing mossy fiber sprouting (*p* < 0.001). All groups were statistically different from each other (post hoc tests *p* < 0.01), Figure 3A. A correlation was obtained between the degree of mossy fiber sprouting and ISI for granule cells (correlations coefficient R = −0.95, *p* < 0.0001). The mean values (standard deviation, SD) and 95% CI for mossy cell ISI in the control group and percentage 10–50% of mossy fiber sprouting groups were 23.5 (1.3) ms (95% CI, 22.8–24.2) vs. 14.4 (0.6) ms (95% CI, 14.0–14.7) vs. 12.3 (0.5) ms (95% CI, 12.0–12.6) vs. 10.9 (0.4) ms (95% CI, 10.7–11.2) vs. 9.4 (0.3) ms (95% CI, 9.2–9.5) vs. 8.2 (0.2) ms (95% CI, 8.1–8.3). There were statistically significant differences in mossy cell ISI versus groups (*p* < 0.01). All groups were statistically different from each other (post hoc tests *p* < 0.001), Figure 3B. A correlation was obtained between the degree of mossy fiber sprouting and ISI for mossy cells (correlations coefficient R = −0.99, *p* < 0.0001). The mean values (standard deviation, SD) and 95% CI for basket cell ISI in the control group and percentage 10–50% of mossy fiber sprouting groups were 12.5 (0.2) ms, (95% CI, 12.3–12.7) vs. 10.3 (0.2) ms, (95% CI, 10.1–10.4) vs. 9.5 (0.2) ms, (95% CI, 9.3–9.7) vs. 8.9 (0.2) ms, (95% CI, 8.7–9.1) vs. 8.1 (0.2) ms, (95% CI, 7.9–8.3) vs. 7.5 (0.2) ms, (95% CI, 7.3–7.6). There were statistically significant differences in basket cell ISI versus groups (*p* < 0.0001). All groups were statistically different from each other (post hoc tests *p* < 0.001), Figure 3C. A correlation was obtained between the degree of mossy fiber sprouting and ISI for basket cells (correlations coefficient R = −0.99, *p* < 0.0001).

In the control model for hilar perforant path-associated cell the ISI value was 23.2 (0.8) ms and (95% CI, 22.4–24.0), but in the increase in mossy fiber sprouting were 15.9 (0.3) ms and (95% CI, 15.6–16.2), 13.7 (0.2) ms and (95% CI, 13.5–13.8), 12.3 (0.2) ms and (95% CI, 12.1–12.4), 10.6 (0.2) ms and (95% CI, 10.5–10.8). 9.3 (0.2) ms and (95% CI, 9.1–9.5). There were statistically significant differences in hilar perforant path-associated cell ISI versus groups (*p* < 0.0001). Post hoc tests showed that hilar perforant path-associated cell ISI were significantly higher in the control group relative to 30–50% mossy fiber sprouting groups (*p* < 0.05). Furthermore, post hoc tests showed that hilar perforant path-associated cell ISI were significantly higher in 10% mossy fiber sprouting groups, relative to 40–50% mossy fiber sprouting group (*p* < 0.05) and were significantly higher in 20% mossy fiber sprouting group, relative to 50% mossy fiber sprouting group (*p* = 0.0463), Figure 3D. A correlation was obtained between the degree of mossy fiber sprouting and ISI for hilar perforant path-associated cells (correlations coefficient R = −0.99, *p* < 0.0001).

**Figure 4:**
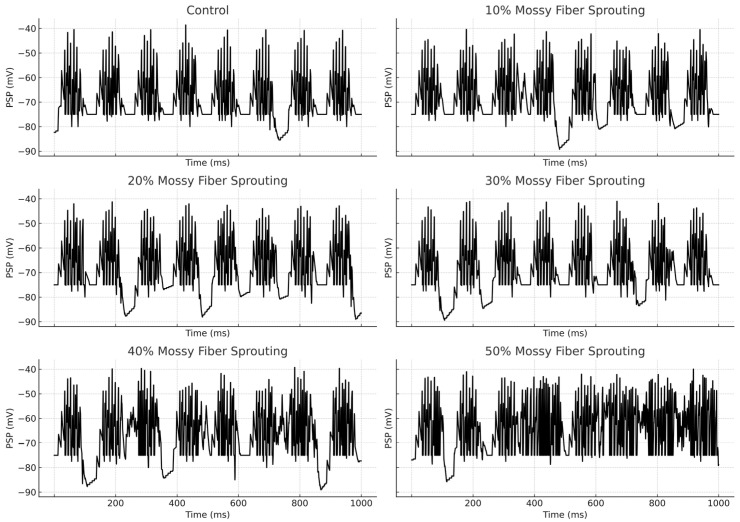
Granule cell membrane voltage traces (PSP—postsynaptic potential) during the first second of the simulation.

An increase in mossy fiber sprouting resulted in an overall rise in mossy cell activity. However, the activation of individual cells was variable—not all responded in the same way. Sprouting promoted more frequent firing, but also amplified the differences between individual cells, Figure 4.

**Figure 5:**
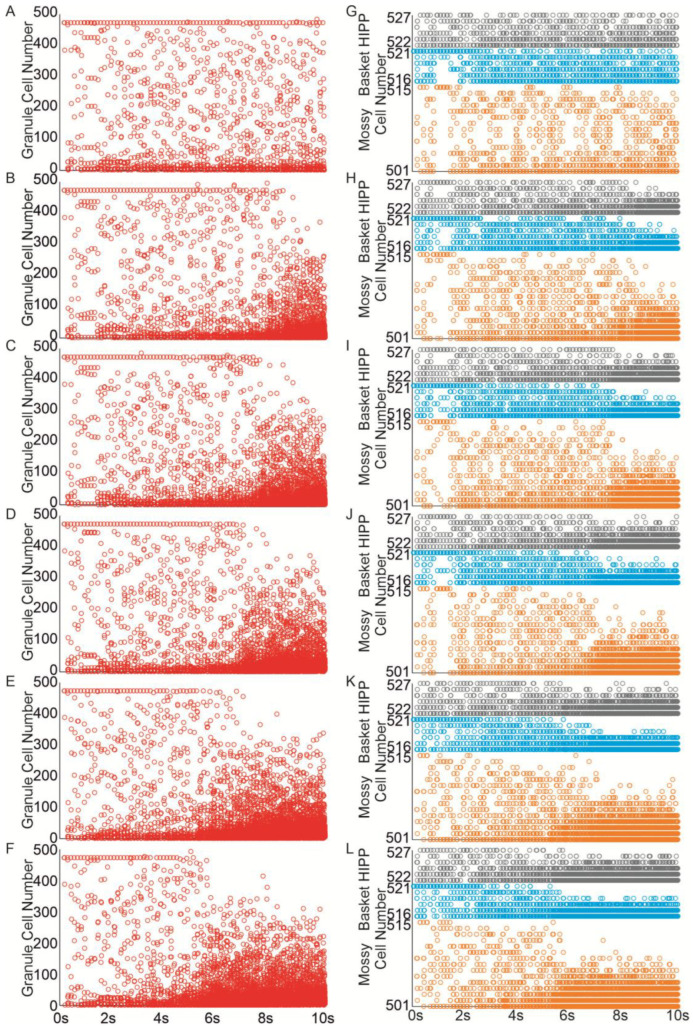
Mossy fiber sprouting increases excitability in the dentate network. (**A**–**F**) Spike raster plots illustrating granule cell activity in the networks in response to perforant path input stimulation ((**A**)—control, (**B**–**F**) mossy fiber sprouting groups 10–50%). (**G**–**L**) Spike raster plots illustrating mossy, basket and hilar perforant path-associated cells activity in the networks in response to perforant path input stimulation ((**G**)—control, (**H**–**L**) mossy fiber sprouting groups 10–50%) (red—granule cell, orange—mossy cell, blue—basket cell, gray—hilar perforant path-associated cell).

The extent of recurrent mossy fiber sprouting gradually increased from 0 to 50%. Even a 10% increase (equivalent to adding 10 recurrent mossy fiber connections per granule cell) led to the spread of activity from the initially activated granule cells to others, Figure 5B, and interneurons, Figure 5H. Increasing mossy fiber sprouting to 25% and 50% resulted in a more extensive propagation of activity, leading to the activation of all granule cells in the network and an extended duration of stimulation-induced network activity Figure 5A–F. When mossy fiber sprouting reached 15%, the localized activity extended to all inhibitory cells within the network Figure 5I–L.

### 3.2. The Role of Pathological Phenomena of Mossy Fiber Sprouting and Mossy Cell Loss

**Figure 6:**
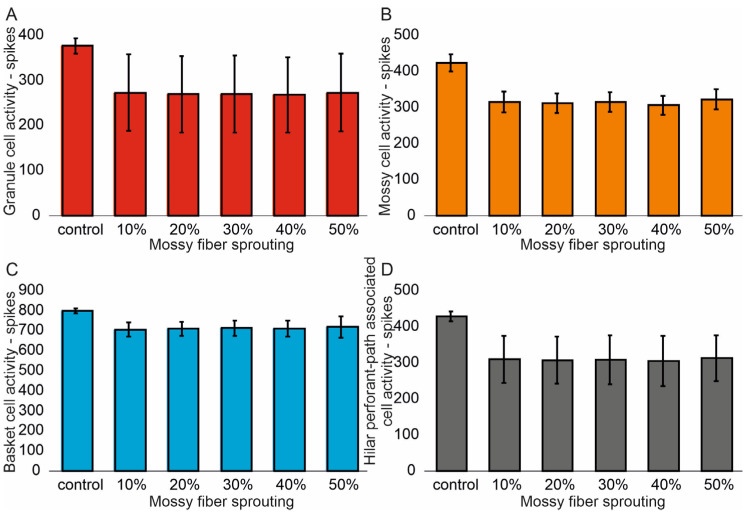
The cell activity (spikes) in the control group and mossy fiber sprouting groups and 50% mossy cell loss. (**A**) Granule cell activity. (**B**) Mossy cell activity. (**C**) Basket cell activity. (**D**) Hilar perforant path-associated cell activity.

The mean values (standard deviation, SD) and 95% CI for granule cell activity in the control group and percentage 10–50% of mossy fiber sprouting and 50% mossy cell loss groups are 376.4 (16.7) (95% CI, 374.9–377.8) vs. 273.3 (84.1) (95% CI, 265.9–280.7) vs. 269.4 (84.9) (95% CI, 261.9–276.8) vs. 270.0 (85.7) (95% CI, 262.5–277.5) vs. 268.4 (83.3) (95% CI, 261.1–275.7) vs. 273.1 (86.4) (95% CI, 265.5–280.7). There were statistically significant differences in granule cell activity versus groups (*p* < 0.001). Post hoc tests showed that granule cell activity was significantly higher in the control group relative to mossy fiber sprouting and mossy cell loss groups (*p* < 0.01), Figure 6A. A correlation was obtained between the degree of mossy fiber sprouting (50% mossy cell loss) and the number of spikes for granule cells (correlations coefficient R = −0.40, *p* < 0.0001). The mean values (standard deviation, SD) and 95% CI for mossy cell activity in the control group and percentage 10–50% of mossy fiber sprouting and 50% mossy cell loss groups are 424.5 (23.7) (95% CI, 411.4–437.7) vs. 315.6 (28.7) (95% CI, 289.1–342.1) vs. 312.0 (27.1) (95% CI, 286.9–337.1) vs. 314.9 (27.2) (95% CI, 289.7–340.0) vs. 306.3 (26.6) (95% CI, 281.7–330.9) vs. 322.6 (28.0) (95% CI, 296.7–348.4). There were statistically significant differences in mossy cell activity versus groups (*p* < 0.0001). Post hoc tests showed that mossy cell activity was significantly higher in the control group, relative to mossy fiber sprouting and mossy cell loss groups (*p* < 0.05), Figure 6B. A correlation was obtained between the degree of mossy fiber sprouting (50% mossy cell loss) and the number of spikes for mossy cells (correlations coefficient R = −0.61, *p* < 0.0001).

The mean values (standard deviation, SD) and 95% CI for basket cell activity in the control group and percentage 10–50% of mossy fiber sprouting and 50% mossy cell loss groups are 799.3 (12.2) (95% CI, 786.5–812.2) vs. 706.2 (34.0) (95% CI, 670.5–741.9) vs. 710.0 (35.1) (95% CI, 673.2–746.8) vs. 712.7 (37.8) (95% CI, 673.0–752.3) vs. 710.5 (40.0) (95% CI, 668.5–752.5) vs. 718.8 (52.9) (95% CI, 663.3–774.4). There were statistically significant differences in basket cell activity versus groups (*p* = 0.0119). Post hoc tests showed that mossy cell activity was significantly higher in the control group relative to 10% mossy fiber sprouting and 50% mossy cell loss group (*p* = 0.0151) and 40% mossy fiber sprouting and 50% mossy cell loss group (*p* = 0.0338), Figure 6C. There was no statistically significant correlation between the degree of mossy fiber sprouting (50% mossy cell loss) and the number of spikes for basket cells (correlations coefficient R = −0.31, *p* = 0.0649). The mean values (standard deviation, SD) and 95% CI for hilar perforant path-associated cell activity in the control group and percentage 10–50% of mossy fiber sprouting and 50% mossy cell groups are 429.3 (13.5) (95% CI, 415.2–443.5) vs. 309.5 (65.7) (95% CI, 240.6–378.4) vs. 307.7 (65.2) (95% CI, 239.2–376.1) vs. 309.0 (67.5) (95% CI, 238.2–379.8) vs. 305.0 (69.2) (95% CI, 232.4–377.6) vs. 313.2 (63.1) (95% CI, 247.0–379.3). There were statistically significant differences in hilar perforant path-associated cell activity versus groups (*p* = 0.0114). Post hoc tests showed that hilar perforant path-associated cell activity were significantly higher in the control group relative to 20–40% mossy fiber sprouting and 50% mossy cell loss groups (*p* < 0.05), Figure 6D. A correlation was obtained between the degree of mossy fiber sprouting (50% mossy cell loss) and the number of spikes for hilar perforant path-associated cells (correlations coefficient R = −0.41, *p* = 0.0123).

**Figure 7:**
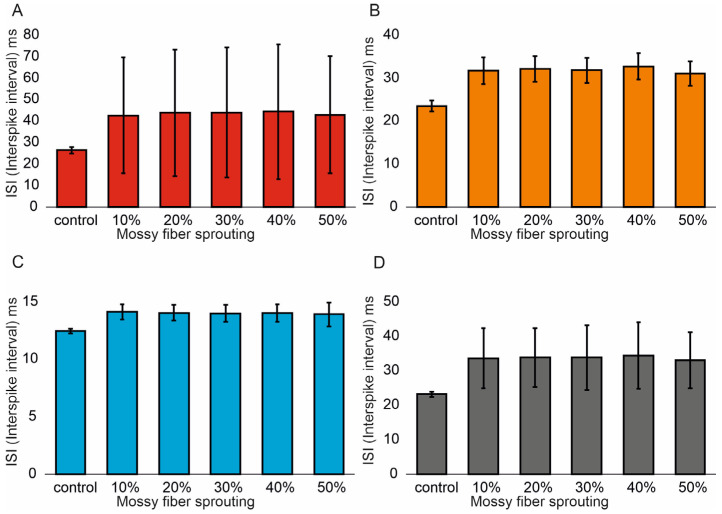
Interspike interval in the control group and mossy fiber sprouting groups and 50% mossy cell loss. (**A**) Granule cell activity. (**B**) Mossy cell activity. (**C**) Basket cell activity. (**D**) Hilar perforant path-associated cell activity.

In the control model for granule cells, the ISI value was 26.5 (1.4) ms and (95% CI, 26.4–26.6). In contrast, with increased mossy fiber sprouting and 50% mossy cell loss, the values were 42.7 (27.1) ms and (95% CI, 40.4–45.1), 43.9 (29.4) ms and (95% CI, 41.3–46.5), 44.0 (30.3) ms and (95% CI, 41.4–46.7), 44.4 (31.3) ms and (95% CI, 41.7–47.2), and 43.0 (27.3) ms and (95% CI, 40.6–45.4). There were statistically significant differences in ISI for granule cells under increased mossy fiber sprouting and 50% mossy cell loss (*p* < 0.001). Post hoc tests showed that granule cell ISI were significantly lower in the control group relative to mossy fiber sprouting and mossy cell loss groups (*p* < 0.01), Figure 7A. A correlation was obtained between the degree of mossy fiber sprouting (50% mossy cell loss) and ISI for granule cells (correlations coefficient R = 0.40, *p* < 0.0001).

The mean values (standard deviation, SD) and 95% CI for mossy cel ISI in the control group and percentage 10–50% of mossy fiber sprouting and 50% mossy cell loss groups are 23.5 (1.3) ms (95% CI, 22.8–24.2) vs. 31.8 (3.1) ms (95% CI, 28.9–34.6) vs. 32.1 (3.0) ms (95% CI, 29.3–34.9) vs. 31.8 (3.0) ms (95% CI, 29.1–34.6) vs. 32.7 (3.0) ms (95% CI, 29.9–35.5) vs. 31.1 (2.9) ms (95% CI, 28.4–33.7). There were statistically significant differences in mossy cell ISI versus groups (*p* < 0.0001). Post hoc tests showed that mossy cell ISI in 20–40% mossy fiber sprouting and 50% mossy cell were significantly higher relative to the control group (*p* < 0.05), Figure 7B. A correlation was obtained between the degree of mossy fiber sprouting (50% mossy cell loss) and ISI for mossy cells (correlations coefficient R = 0.60, *p* < 0.0001). The mean values (standard deviation, SD) and 95% CI for basket cell ISI in the control group and percentage 10–50% of mossy fiber sprouting and 50% mossy cell groups are 12.5 (0.2) ms, (95% CI, 12.3–12.7) vs. 14.1 (0.7) ms, (95% CI, 13.4–14.8) vs. 14.1 (0.7) ms, (95% CI, 13.3–14.8) vs. 14.0 (0.7) ms, (95% CI, 13.2–14.8) vs. 14.1 (0.8) ms, (95% CI, 13.3–14.9) vs. 13.9 (1.1) ms, (95% CI, 12.8–15.0). There were statistically significant differences in basket cell ISI versus groups (*p* = 0.0116). Post hoc tests showed that basket cell ISI were significantly lower in the control group, relative to 10% mossy fiber sprouting and 50% mossy cell loss group (*p* = 0.0137) and 40% mossy fiber sprouting and 50% mossy cell loss group (*p* = 0.0322), Figure 7C. There was no statistically significant correlation between the degree of mossy fiber sprouting (50% mossy cell loss) and ISI for basket cells (correlations coefficient R = −0.31, *p* = 0.0658). In the control model for hilar perforant path-associated cells the ISI value was 23.2 (0.8) ms and (95% CI, 22.4–24.0), but in the increase in mossy fiber sprouting and 50% mossy cell were 33.7 (8.7) ms and (95% CI, 24.6–42.7), 33.8 (8.5) ms and (95% CI, 24.9–42.8), 33.9 (9.4) ms and (95% CI, 24.0–43.7), 34.4 (9.7) ms and (95% CI, 24.3–44.6). 33.1 (8.1) ms and (95% CI, 24.6–41.7). There were statistically significant differences in hilar perforant path-associated cell ISI versus groups (*p* = 0.0114). Post hoc tests showed that hilar perforant path-associated cell ISI were significantly lower in the control group, relative to 10% mossy fiber sprouting and 50% mossy cell loss group (*p* = 0.0353) and 40% mossy fiber sprouting and 50% mossy cell loss group (*p* = 0.0244), Figure 7D. A correlation was obtained between the degree of mossy fiber sprouting (50% mossy cell loss) and ISI for hilar perforant path-associated cells (correlations coefficient R = 0.41, *p* = 0.0122).

**Figure 8:**
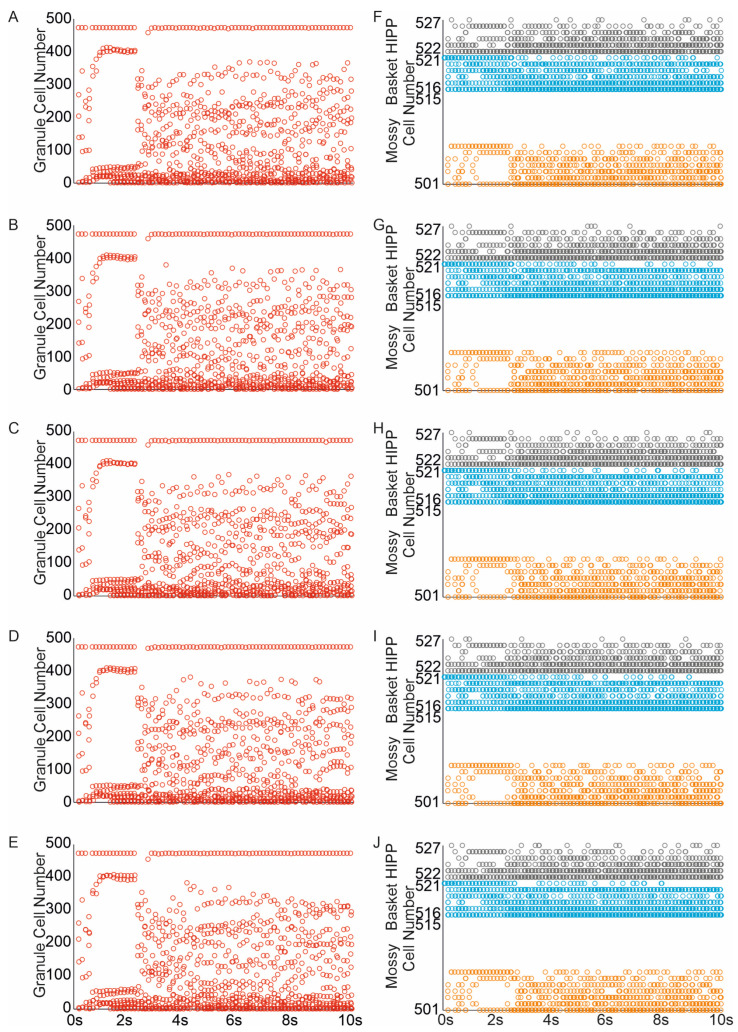
Impact of mossy cell loss and sprouting on dentate network excitability. (**A**–**E**) Spike raster plots illustrating granule cell activity in the networks in response to perforant path input stimulation ((**A**–**E**) mossy cell loss and sprouting groups 10–50%). (**F**–**J**) Spike raster plots illustrating mossy, basket and hilar perforant path-associated cells activity in the networks in re-sponse to perforant path input stimulation ((**F**–**J**) mossy cell loss and sprouting groups 10–50%) (red—granule cell, orange—mossy cell, blue—basket cell, gray—hilar perforant path-associated cell).

The random removal of eight mossy cells (representing 50% of the mossy cell population, similar to the extent of mossy cell loss observed after moderate head trauma) led to a reduction in both the average firing rate of all cells and the number of granule cells and interneurons engaged in network activity Figure 8A–C,F–J. The corresponding mossy cell spike raster indicated a diminished spread of network activity to mossy cells, likely because the “dead” cells were unable to contribute to the propagation of activity. The complete loss of mossy cells resulted in a further decline in network activity spread, Figure 8D,E.

## 4. Discussion

This study focuses on analyzing the role of two distinct histopathological features common to TLE and head injuries—mossy fiber sprouting and the loss of excitatory hilar cells—in modulating the excitability of the dentate gyrus. In an animal model of epilepsy, irregular axon branches and collaterals extending from a primary axon have been detected within the hilus of the dentate gyrus [42,43,44,45]. My simulation results showed that even a low level of mossy fiber sprouting is sufficient to increase the excitability of the dentate gyrus in response to lamellar activation of the perforant path. In contrast, the removal of mossy cells reduces the excitability of granule cells and inhibits the propagation of network hyperexcitability. This suggests that surviving mossy cells enhance the excitability of the dentate gyrus, even in the absence of changes in their intrinsic or synaptic properties [46,47]. Although the role of abnormal mossy fiber projections remains uncertain, one hypothesis suggests that synapses formed by mossy fiber sprouting are functionally active. This is because axon selection relies on excitatory pre- and postsynaptic activity, leading to hyperexcitation of the dentate gyrus and CA3, which may contribute to the onset of epileptogenesis [48,49,50,51,52]. As abnormal mossy fiber sprouting intensifies, excitation levels rise, and according to the compensation theory, the network attempts to counteract this imbalance. When mossy fiber sprouting reaches a point where it boosts granule cell activity enough to surpass the threshold for non-synaptic mechanisms, the network undergoes a shift. This shift marks the transition to an epileptic state. Essentially, once this threshold is crossed, the network becomes more prone to seizure activity.

My study, as a computational-simulation project, maintains a strong alignment between the model and experimental data. On the other hand, previous computational studies have analyzed the role of recurrent excitatory collaterals in epileptic activity within CA3 [53,54]. Meanwhile, other studies using a biophysically realistic computational model of the dentate gyrus have simulated the loss of smaller cells and the sprouting of smaller fibers [23,55]. My simulations show a correlation between the degree of mossy fiber sprouting and the average number and duration of discharges in granule cells of the dentate gyrus and other cells. These results are generally consistent with experimental observations. Studies have indicated a high degree of mossy fiber sprouting in animals with a higher frequency of epileptic seizures [15,56]. Nevertheless, the existence and strength of the relationship between sprouting and the frequency and duration of seizures in animal models remain subjects of debate. Some studies suggest a strong correlation, while others report more variable findings. The complexity of the underlying mechanisms may contribute to these discrepancies. Further research is needed to clarify the role of mossy fiber sprouting in seizure activity [57]. The simulations also indicated that the spatially restricted, lamellar distribution of sprouted mossy fiber contacts, as described in in vivo studies, was a key factor in sustaining seizure-like activity in the network. This specific pattern of connectivity appears to contribute to persistent hyperexcitability. The findings suggest that localized structural changes can significantly impact network dynamics. Understanding these mechanisms may provide insights into the development of epilepsy.

Finally, although the loss of mossy cells may contribute to dentate gyrus hyperexcitability by promoting mossy fiber sprouting, our simulations show that mossy cell loss alone is neither necessary nor sufficient to increase network excitability [58,59]. Instead, the removal of mossy cells led to a decrease in excitability in the modeled networks [60,61]. This finding aligns with experimental data [47]. These results highlight the crucial role of mossy fiber sprouting, even in cases where only relatively mild sprouting occurs, as seen after a moderate experimental head injury. Even a low level of mossy fiber sprouting can significantly impact network excitability. This suggests that structural changes in the dentate gyrus may contribute to altered neuronal activity. Such findings emphasize the importance of considering even subtle histopathological changes in brain injury models.

## 5. Conclusions

In contrast to the strong impact of mossy fiber sprouting on inducing hyperexcitability, the removal of mossy cells led to a decrease in the granule cell response to perforant path activation, which is consistent with the latest experimental data. These findings highlight the critical role of mossy fiber sprouting, even in cases where only relatively mild sprouting occurs, as seen after a moderate experimental head injury. Even a low level of mossy fiber sprouting can have a significant effect on network excitability. This suggests that structural changes in the dentate gyrus may contribute to altered neuronal activity. Such observations underscore the importance of considering even subtle histopathological changes in brain injury models.

## 6. Limitations

The primary limitation of in silico research lies in understanding the potential of a mathematical and computational model. The predictions and outcomes of these models are influenced by the knowledge and assumptions incorporated during their creation. As a result, the accuracy of the model depends on the quality of the information used in its development. This highlights the importance of continuously updating and refining the underlying assumptions to improve model reliability.

## Figures and Tables

**Table 1:** Physiological properties of cell types.

Physiological Property	Granule Cell	Mossy Cell	Basket Cell	Hilar Perforant Path-Associated Cell
RP, mV	−75	−60	−60	−70
TP, mV	−49	−48	−45	−50
TR, ms	1.5	1.5	1.5	1.5
EPSPa, mV	4.7	4	7.5	6
IPSPa, mV	−5	−6	−4	−6
CaT, mV	−68	−68	x	x

RP—resting potential, TP—threshold potential, TR—time of refrakcion, EPSPa—amplitude of excitatory postsynaptic potential, IPSPa—amplitudę of inhibitory postsynaptic potential, CaT—threshold for the removal of the Mg ion block for NMDA channels, x—not applicable.

## Data Availability

The data presented in this study are available on request from the corresponding author.
